# Supplementary material for: Effect of Brewing Methods on Acrylamide Content and Antioxidant Activity: Studying Eight Different Filter Coffee Preparations
Source: Antioxidants (Basel). 2023 Oct 21;12(10):1888. doi: 10.3390/antiox12101888 (PMC10604660; doi:10.3390/antiox12101888)
Supplement: Supplementary file 1 [file antioxidants-12-01888-s001.zip › antioxidants-2658682-supplementary.pptx]

## Slide 1
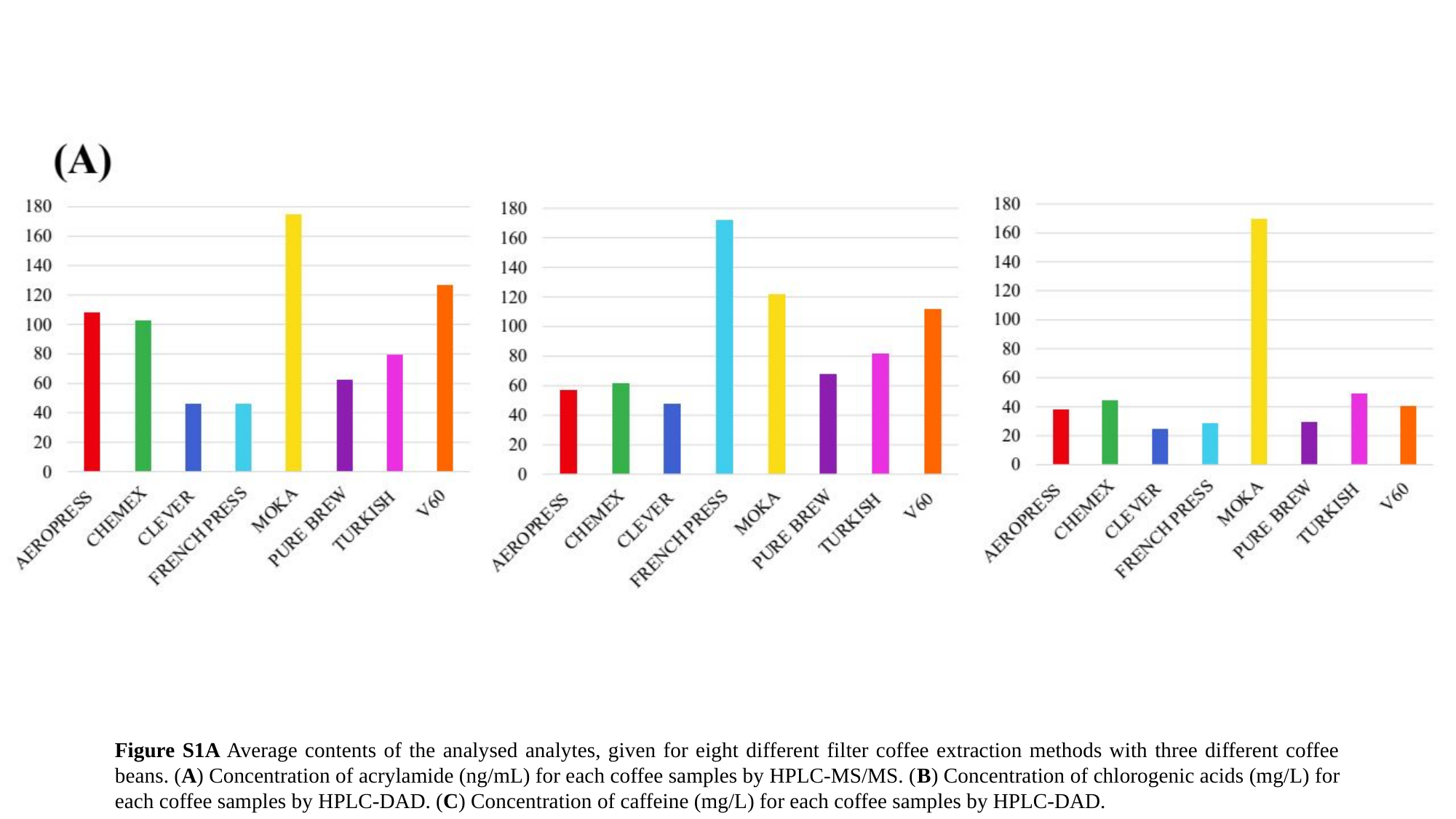

Figure S1A Average contents of the analysed analytes, given for eight different filter coffee extraction methods with three different coffee beans. (A) Concentration of acrylamide (ng/mL) for each coffee samples by HPLC-MS/MS. (B) Concentration of chlorogenic acids (mg/L) for each coffee samples by HPLC-DAD. (C) Concentration of caffeine (mg/L) for each coffee samples by HPLC-DAD.

## Slide 2
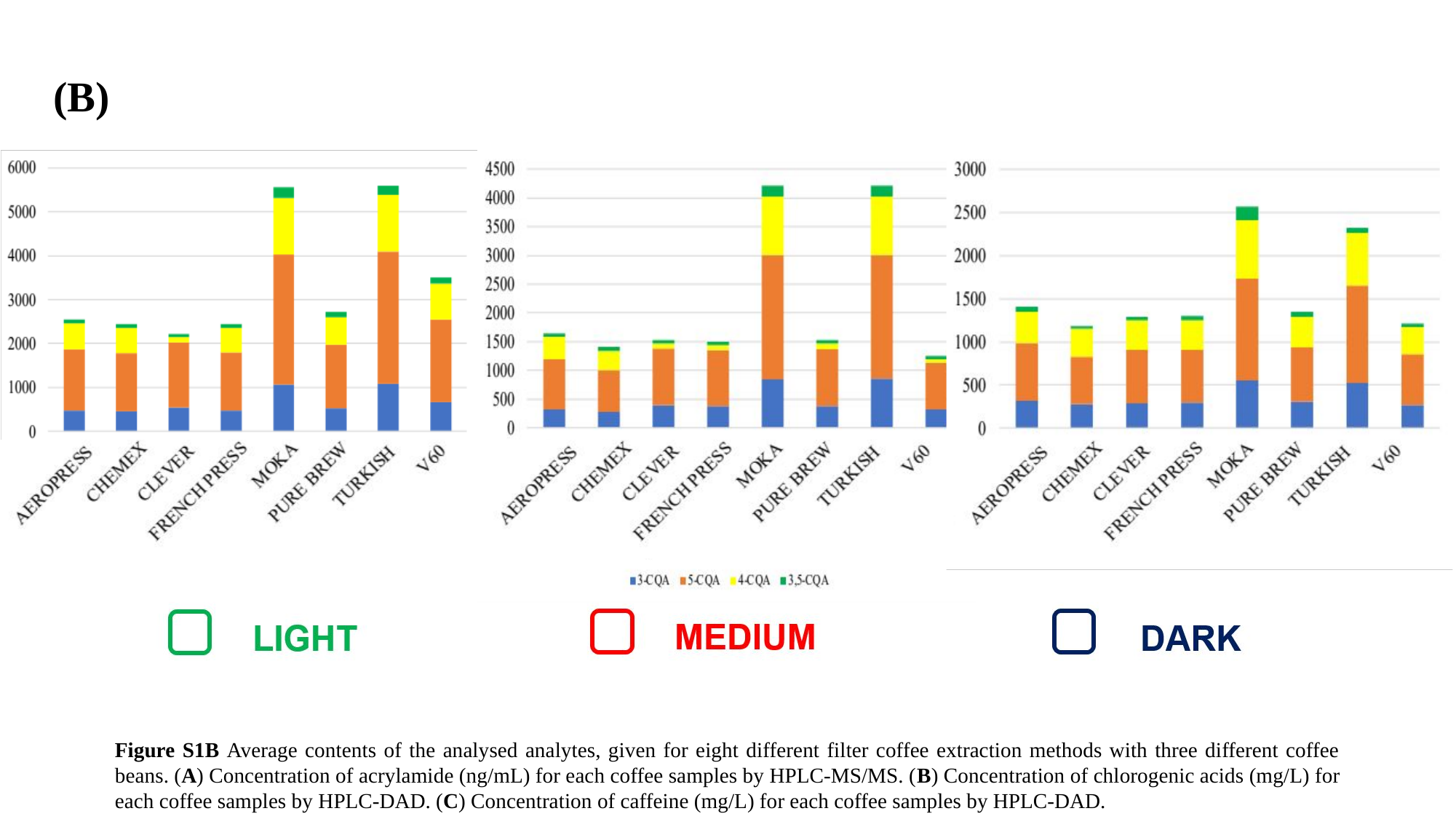

(B)
Figure S1B Average contents of the analysed analytes, given for eight different filter coffee extraction methods with three different coffee beans. (A) Concentration of acrylamide (ng/mL) for each coffee samples by HPLC-MS/MS. (B) Concentration of chlorogenic acids (mg/L) for each coffee samples by HPLC-DAD. (C) Concentration of caffeine (mg/L) for each coffee samples by HPLC-DAD.

## Slide 3
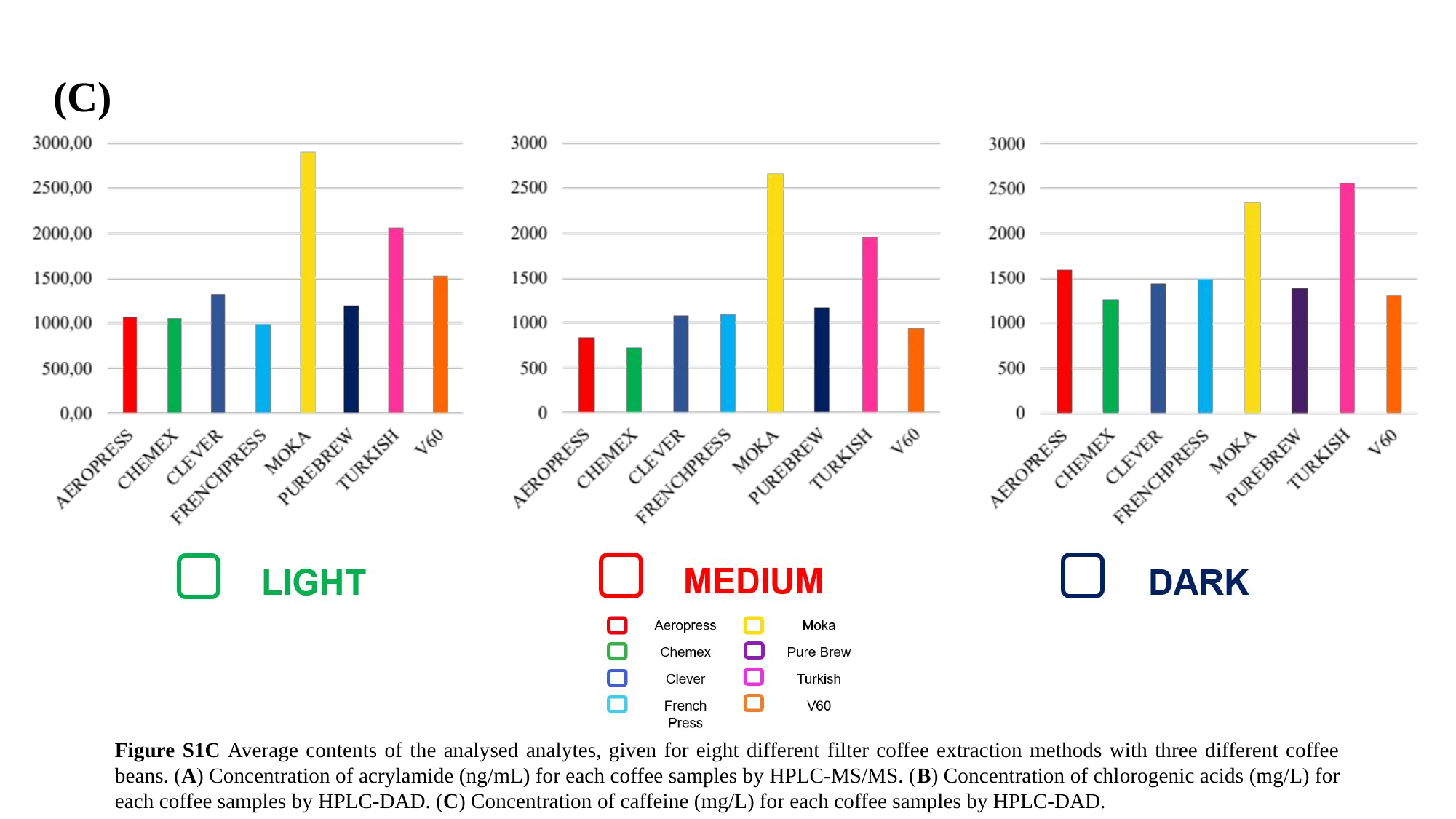

(C)
Figure S1C Average contents of the analysed analytes, given for eight different filter coffee extraction methods with three different coffee beans. (A) Concentration of acrylamide (ng/mL) for each coffee samples by HPLC-MS/MS. (B) Concentration of chlorogenic acids (mg/L) for each coffee samples by HPLC-DAD. (C) Concentration of caffeine (mg/L) for each coffee samples by HPLC-DAD.

## Slide 4
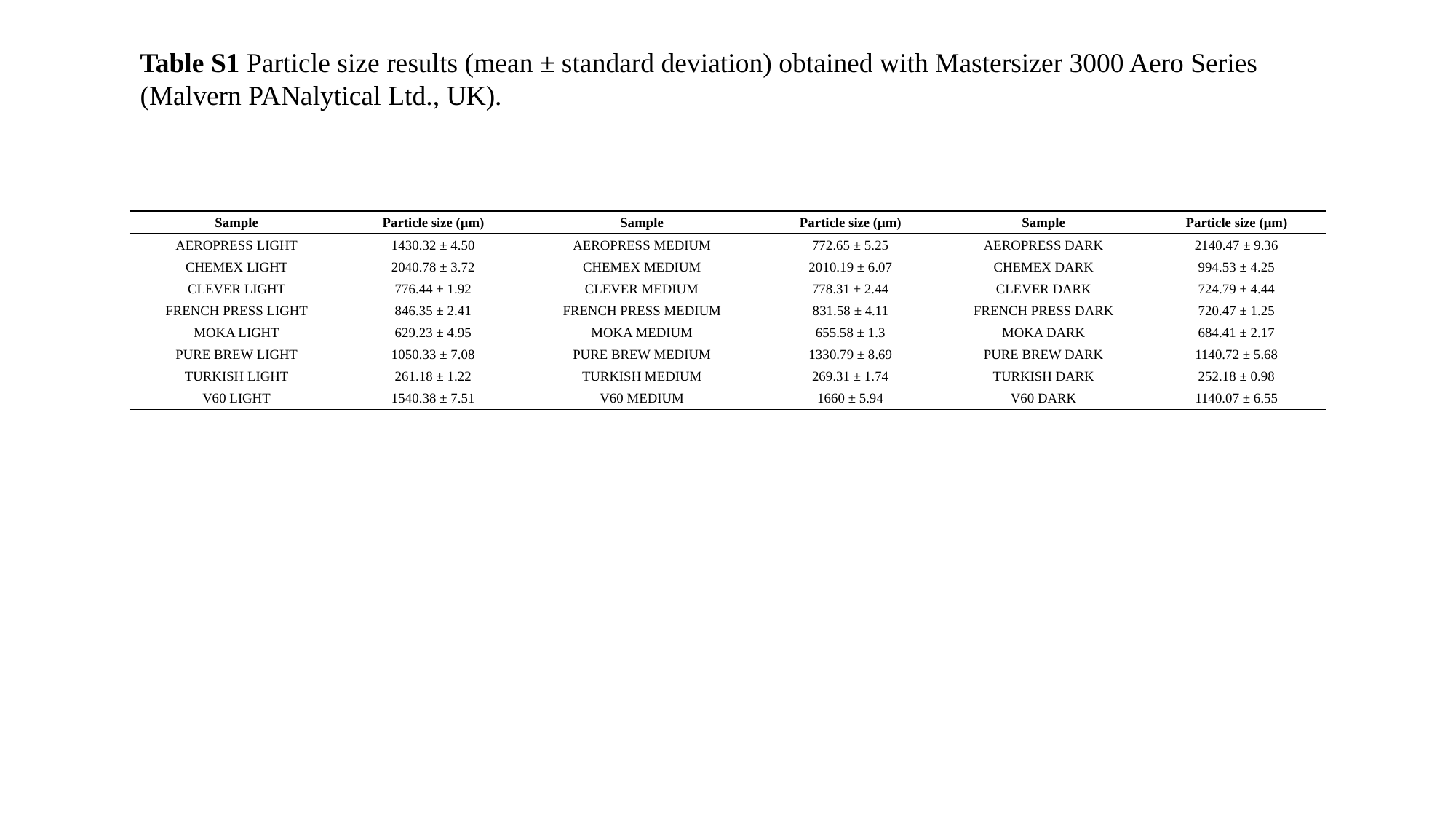

Table S1 Particle size results (mean ± standard deviation) obtained with Mastersizer 3000 Aero Series (Malvern PANalytical Ltd., UK).
| Sample | Particle size (μm) | Sample | Particle size (μm) | Sample | Particle size (μm) |
| --- | --- | --- | --- | --- | --- |
| AEROPRESS LIGHT | 1430.32 ± 4.50 | AEROPRESS MEDIUM | 772.65 ± 5.25 | AEROPRESS DARK | 2140.47 ± 9.36 |
| CHEMEX LIGHT | 2040.78 ± 3.72 | CHEMEX MEDIUM | 2010.19 ± 6.07 | CHEMEX DARK | 994.53 ± 4.25 |
| CLEVER LIGHT | 776.44 ± 1.92 | CLEVER MEDIUM | 778.31 ± 2.44 | CLEVER DARK | 724.79 ± 4.44 |
| FRENCH PRESS LIGHT | 846.35 ± 2.41 | FRENCH PRESS MEDIUM | 831.58 ± 4.11 | FRENCH PRESS DARK | 720.47 ± 1.25 |
| MOKA LIGHT | 629.23 ± 4.95 | MOKA MEDIUM | 655.58 ± 1.3 | MOKA DARK | 684.41 ± 2.17 |
| PURE BREW LIGHT | 1050.33 ± 7.08 | PURE BREW MEDIUM | 1330.79 ± 8.69 | PURE BREW DARK | 1140.72 ± 5.68 |
| TURKISH LIGHT | 261.18 ± 1.22 | TURKISH MEDIUM | 269.31 ± 1.74 | TURKISH DARK | 252.18 ± 0.98 |
| V60 LIGHT | 1540.38 ± 7.51 | V60 MEDIUM | 1660 ± 5.94 | V60 DARK | 1140.07 ± 6.55 |
